# Conservation of Differential Animal MicroRNA Processing by Drosha and Dicer

**DOI:** 10.3389/fmolb.2021.730006

**Published:** 2022-01-03

**Authors:** Xiaoxiao Zhang, Fanming Yang, Fanzou Liu, Qiuhuan Tian, Min Hu, Peng Li, Yan Zeng

**Affiliations:** Department of Zoology, College of Life Sciences, Nanjing Agricultural University, Nanjing, China

**Keywords:** relative specificity, microRNA, drosha, dicer, secondary structure, differential cleavage

## Abstract

In complex biochemical systems, an enzyme, protein, or RNA, symbolized as E, has hundreds or thousands of substrates or interacting partners. The relative specificity hypothesis proposes that such an E would differentially interact with and influence its many distinct, downstream substrates, thereby regulating the underlying biological process (es). The importance of relative specificity has been underappreciated, and evidence of its physiological consequences particularly lacking. Previously we showed that human Drosha and Dicer ribonucleases (RNases) both discriminate their respective microRNA (miRNA) substrates, and that differential cleavage by Drosha contributes to global differential miRNA expression. If relative specificity is an important biological mechanism, it should be evolutionarily conserved. To test this hypothesis, we hereby examined the cleavage of hundreds of zebrafish and fruitfly miRNA intermediates by Drosha and Dicer and the impact on miRNA biogenesis in these organisms. We showed that Drosha action regulates differential miRNA expression in zebrafish and fruitflies and identified the conserved secondary structure features and sequences in miRNA transcripts that control Drosha activity and miRNA expression. Our results established the conservation of miRNA processing mechanisms and regulatory functions by Drosha and Dicer, greatly strengthened the evidence for the physiological consequences of relative specificity as well as demonstrated its evolutionary significance.

## Introduction

The relative specificity hypothesis of complex biochemical systems posits that an E differentially interacts with and/or impacts its many, e.g., 100 or more, substrates or interacting partners, and, hence, exerts a critical regulatory function in the background (Zeng, 2011). Although that an E discriminates its diverse substrates is intuitive, explicit, large-scale evidence is scarce in the literature, even rarer is the direct demonstration of biological relevance. In the field of transcriptional regulation the concept of “quantitative continua” has been formulated (Biggin, 2011). DF proteins control the degradation of m^6^A-containing mRNAs by the number of m^6^A sites they bind to on diverse mRNAs (Zaccara and Jaffrey, 2020). A salient example was demonstrated by Swaffer et al. (2016) that the fission yeast CDK has substrate preferences, phosphorylating the good substrates early to promote DNA replication, and the poor ones later to promote cytokinesis. If CDK phosphorylates the poor substrates prematurely, cells will divide without DNA doubling and subsequently die, demonstrating the functional consequence of relative specificity. In other words, CDK regulates cell cycle progression not only by phosphorylating myriad substrates, but also by selectively phosphorylating them at different times due to relative specificity. Despite these sporadic examples and a few others, however, only in recent years with the advent of genomics techniques have people begun to investigate relative specificity and its regulation of complex systems (Zeng, 2011; Zeng, 2014).

We have used human miRNAs as a model to study relative specificity. There are hundreds of miRNAs in humans, and each miRNA regulates the expression of hundreds of target genes (Bartel, 2018). During animal miRNA processing, Drosha complexed with DGCR8 (Pasha in *Drosophila melanogaster*) cleaves primary miRNA transcripts (pri-miRNAs) to generate precursor miRNAs (pre-miRNAs), which are then cleaved by Dicer to produce miRNA duplex intermediates (Billy et al., 2001; Grishok et al., 2001; Hutvágner et al., 2001; Ketting et al., 2001; Lee et al., 2003; Denli et al., 2004; Gregory et al., 2004; Han et al., 2004; Landthaler et al., 2004). We showed that human Drosha/DGCR8 cleaves hundreds of pri-miRNAs with different efficiencies *in vitro*, which correlates with mature miRNA expression *in vivo*, thus revealing a new, regulatory role by Drosha (Feng et al., 2011). Dicer also discriminates against pre-miRNAs, albeit without a significant correlation to miRNA expression (Feng et al., 2012). An explanation is that as Drosha acts upstream of Dicer in the same pathway, the selectivity of Drosha may be the dominant, or rate-limiting regulatory mechanism (Zeng, 2014). Of note, this level of regulation is distinct from the production of miRNA isoforms, which results from subtle variations in the RNA structures and their interactions with the RNases (Bartel, 2018). miRNA function likewise exhibits relative specificity, as miRNAs such as miR-124 inhibit different target genes to various extent, potentially contributing to mRNA differential expression in humans (Li et al., 2019a). Targeting efficacy by miRNAs had also been examined using large-scale artificial target libraries, although its functional relevance to endogenous gene expression was unexplored in those reports (Slutskin et al., 2018; Becker et al., 2019; McGeary et al., 2019).

Our work on relative specificity and miRNAs inspired a number of questions this study aimed to address. The first was that only human Drosha and Dicer have been investigated extensively for their activities, while little is known about their mechanisms in other animals, or whether the RNA requirements are the same. Despite the assumption that all animal pri-miRNAs are alike, species-specific processing has been suggested for *Caenorhabditis elegans* and, plausibly, *Drosophila melanogaster* (Auyeung et al., 2013), pointing to a need to investigate miRNA biogenesis in organisms other than humans. The second was that by examining differential miRNA processing in other species we might be able to provide additional evidence for relative specificity and its physiological consequences. Lastly, and the most importantly, if relative specificity is a general principle, it should be conserved through evolution. For example, do the zebrafish (*Danio rerio*) and fruitfly (*Drosophila melanogaster*) Drosha and Dicer also selectively process their miRNA substrates, and crucially, does the differential cleavage by Drosha, a proxy for relative specificity, play a preeminent role in regulating miRNA expression in the animal kingdom, as seen in humans (Zeng, 2014)? To answer these questions, therefore, we prepared recombinant zebrafish and fruitfly Dicer and Drosha enzymes and conducted large-scale processing reactions *in vitro*, and then correlated the results to miRNA expression in the respective organisms.

## Materials and Methods

### Molecular Cloning

Plasmids expressing the zebrafish Dicer, Drosha, and DGCR8 had been described (Li et al., 2019b). To clone the fruitfly proteins, total adult fruitfly RNA was acquired from the Shanghai Institute of Biochemistry and Cell Biology (Shanghai, China), reverse-transcribed using a first-strand cDNA synthesis kit (Invitrogen, Carlsbad, CA, United States) to produce cDNA, which was then used in PCR amplification by Phusion DNA Polymerase (New England BioLabs, Ipswich, MA, United States). Dicer (CG4792, Dcr-1) cDNA was amplified using primers 5′-GCA​AGC​TTA​TGG​CGT​TCC​ACT​GGT​G-3′ and 5′-GCG​AAT​TCT​TAT​TCG​ACC​ATA​GAC​AAT​CT-3′, the resulting PCR product digested with HindIII and EcoRI (New England BioLabs) and inserted into p3xFLAG-CMV 7.1 (Sigma, St Louis, MO, United States) to produce p3xFLAG-CMV-fly Dicer. Drosha (CG8730) was amplified with primers 5′-GCA​AGC​TTA​TGT​ACC​AGC​CGC​CTT​T-3′ and 5′-GCT​CTA​GAT​CAT​CCC​AGC​GAA​GAT​TT-3′, PCR product digested with HindIII and XbaI (New England BioLabs), and inserted into p3xFLAG-CMV 7.1 to produce p3xFLAG-CMV-fly Drosha. Pasha (CG 1800, DGCR8 hereafter for consistency) was amplified using primers 5′-GCG​GAT​CCA​TGG​CGG​AGA​AGC​CGC-3′ and 5′-CGG​CGG​CCG​CTC​AAA​GTT​CCA​CGT​TGT​T-3′, PCR product digested with BamHI and NotI (New England BioLabs), and inserted into pKMyc (Addgene, Watertown, MA, United States), to yield pKMyc-fly DGCR8. Sanger sequencing (Sangon, Shanghai, China) verified identities of the clones. pET28a-HisLoqs-PB, which expresses the His-tagged fruitfly Loqs-PB or R3D1-L protein, a critical co-factor of fly Dicer (Forstemann et al., 2005; Jiang et al., 2005; Saito et al., 2005), was a gift by Dr Qinghua Liu (University of Texas Southwest Medical Center at Dallas).

### Cell Culture

Human 293T cells (Sangon) were maintained at 37°C and 5% CO_2_ in Dulbecco’s modified Eagle’s medium supplemented with 10% fetal bovine serum and 2 mM L-glutamine (Invitrogen). Cells seeded in 6-well plates or p100 dishes (Sangon) were transfected using Lipofectamine 2000 (Invitrogen) and harvested 48–72 h later.

### Protein Purification

Recombinant FLAG-Drosha/Myc-DGCR8 and FLAG-Dicer proteins were produced by (co-)transfecting plasmids into 293T cells, isolated, and analyzed, as described (Li et al., 2019b). To prepare His-Loqs-PB, BL21 cells transformed with pET28a-HisLoqs-PB were induced, lysed, and His-Loqs-PB isolated using the Ni-NTA beads, per manufacture’s instructions (Qiagen, Hilden, Germany). Eluted fractions were dialyzed against 20 mM Tris-HCl pH 7.5, 1 mM EDTA, 1 mM DTT, and 10% glycerol at 4oC overnight. All proteins were stored at −20°C until use.

### Preparation of miRNA Substrates

miRNA genomic sequence information was obtained from miRBase (Kozomara et al., 2019). DNA templates for the synthesis of pri-miRNAs were amplified from zebrafish and fruitfly genomic DNAs (Shanghai Institute of Biochemistry and Cell Biology) by PCR, with one of the primers containing the T7 promoter sequence at its 5′ end. We designed the templates such that the resulting pri-miRNAs would possess approximately 30 extra nucleotides (nt) 5′ and 40 nt 3′ of the pre-miRNAs, as extended sequences are necessary for human Drosha processing (Zeng and Cullen, 2005). For pre-miRNAs, overlapping primers were designed, and DNA templates synthesized by PCR, according to Feng et al. (2012). Because the T7 RNA polymerase transcribes from a G residue, if the 5′ end of a pre-miRNA is not G, in our substrate we would change it to G and modify the corresponding residue near the 3’ end, to maintain the predicted secondary structure (Feng et al., 2012). Primer sequences are listed in Supplementary Tables S1–S4. RNA substrates were then prepared by *in vitro* transcription (Promega, Madison, WI, United States) in the presence of [α-^32^P] CTP (PerkinElmer, Waltham, MA, United States).

### miRNA Processing Assays

RNA processing assays were performed as described (Feng et al., 2011; Feng et al., 2012). In the processing reactions, both Dicer and Drosha proteins were used at approximately 1 ng/μl, and the fruitfly Dicer was supplemented with His-Loqs-PB at approximately 5 ng/μl. Proteins were mixed with ^32^P-labeled RNAs and incubated at 37°C for approximately 30–40 min (Dicer) or 60 min (Drosha), unless indicated otherwise, before denaturing gel electrophoresis. For size markers, typically an in-house set of DNAs (200, 110, 62, 46, 31, and 20 nt in length) were labeled at their 5′-ends with [γ-^32^P] ATP (PerkinElmer) by T4 polynucleotide kinase (New England BioLabs). To compare results from experiments performed on separate days, a normalization control RNA was included in every processing experiment. For zebrafish Dicer the control RNA was dre-pre-let-7a-1; zebrafish Drosha: dre-pri-let-7d-2; fruitfly Dicer: dme-pre-let-7; fruitfly Drosha: dme-pri-let-7. After electrophoresis, gels were fixed, and data analyzed by phosphorimaging (GE Healthcare, Chicago, IL, United States). The raw cleavage efficiency or ratio was calculated as the intensities of predicted products divided by the intensities of the products and the remaining full-length substrate, and then divided by the raw cleavage efficiency of the control RNA to obtain the relative cleavage efficiency (Feng et al., 2011). Relative cleavage efficiencies of the control RNAs were set at 100. Most miRNA substrates were tested at least twice to obtain consistent results.

### Fruitfly Small RNA Sequencing

Total fruitfly RNA, isolated from individual adults (Shanghai Institute of Biochemistry and Cell Biology), was subject to small RNA-seq (Sangon). Sample library was prepared using the VAHTSTM Small RNA Library Prep Kit for Illumina (San Diego, CA, United States) according to the manufacturer’s protocol. Briefly, adaptors were ligated sequentially to the 3′ and 5′ ends of RNAs, universal cDNA synthesis, library amplification and library purification were performed. HiSeq single-end sequencing was carried out according to standard Illumina protocols. Cutadapt (version 1.14) was used to clip the adaptor, and trimmomatic software implemented to remove low-quality base at both ends and filter the raw reads. Qualified reads after filtering low-quality data were analyzed using miRDeep2 software (Friedländer et al., 2008) for aligning reads to miRBase (Kozomara et al., 2019). RNA-seq data have been deposited in the Gene Expression Omnibus under the accession number GSE163852.

### Statistics, Pri-miRNA Secondary Structure Prediction and Sequence Motif Analyses

GraphPad Prism 5.0 (GraphPad Software, San Diego, CA, United) and SPSS 13.0 (IBM, Armonk, NY, United States) were used for the Spearman rank correlation test and Mann-Whitney U test (two-tailed). Fruitfly miRNA expression data were from GSE163852. Zebrafish miRNA expression was retrieved from GSE57169, which included data from zebrafish embryos and a number of adult organs. Information of miRNA expression in 20 human tissues was likewise acquired from public datasets (Supplementary Table S5). Normalized expression values of individual miRNAs from diverse zebrafish or human tissues were combined to reduce cell-specific effects. The expression level of a miRNA was calculated as the sum of the sequence reads for the corresponding miRNA-5p and miRNA-3p (Feng et al., 2011). For secondary structure prediction, the actual pri-miRNA and pre-miRNA substrates (listed in Supplementary Tables S1–S4) were folded using Mfold under default conditions, and the most stable conformations were recorded for further examination (Mathews et al., 1999; Zuker, 2003). Gibbs energy (ΔG) of the terminal loop region was calculated from the pri-miRNA structural prediction, with the terminal loop region starts from the first nucleotide after the 3′ end of the actual or predicted miRNA-5p at the 5′ arm of the hairpin and ends at its corresponding nucleotide at the 3′ arm (Feng et al., 2011). For ΔG of the miRNA duplex region, a pre-miRNA was folded using Mfold, and its ΔG subtracted by that of the terminal loop region. ΔG for the proximal and distal domains of the pre-miRNA-flanking region was analogously computed. The proximal domain contains 12 nt extensions beyond the 5′ and 3′ ends of a pre-miRNA (Feng et al., 2011). There are inherent heterogeneities in the 5′ and 3′ ends of mature miRNAs, and the secondary structure predictions give only estimates.

The presence of sequence motifs (UG, UGU, mGHG, and CNNC) in pri-miRNAs were evaluated as described (Auyeung et al., 2013; Fang and Bartel, 2015; Kwon et al., 2019). mGHG scores were calculated according to Kwon et al. (2019). All zebrafish and fruitfly miRNAs were considered, so were human miRNAs with a name/number lower than 1,000, as the later a human miRNA was discovered or annotated, the less likely it has been characterized as a genuine or canonical miRNA (Feng et al., 2011).

## Results

### Preparation of Recombinant Fruitfly Dicer and Drosha RNases

**FIGURE 1:**
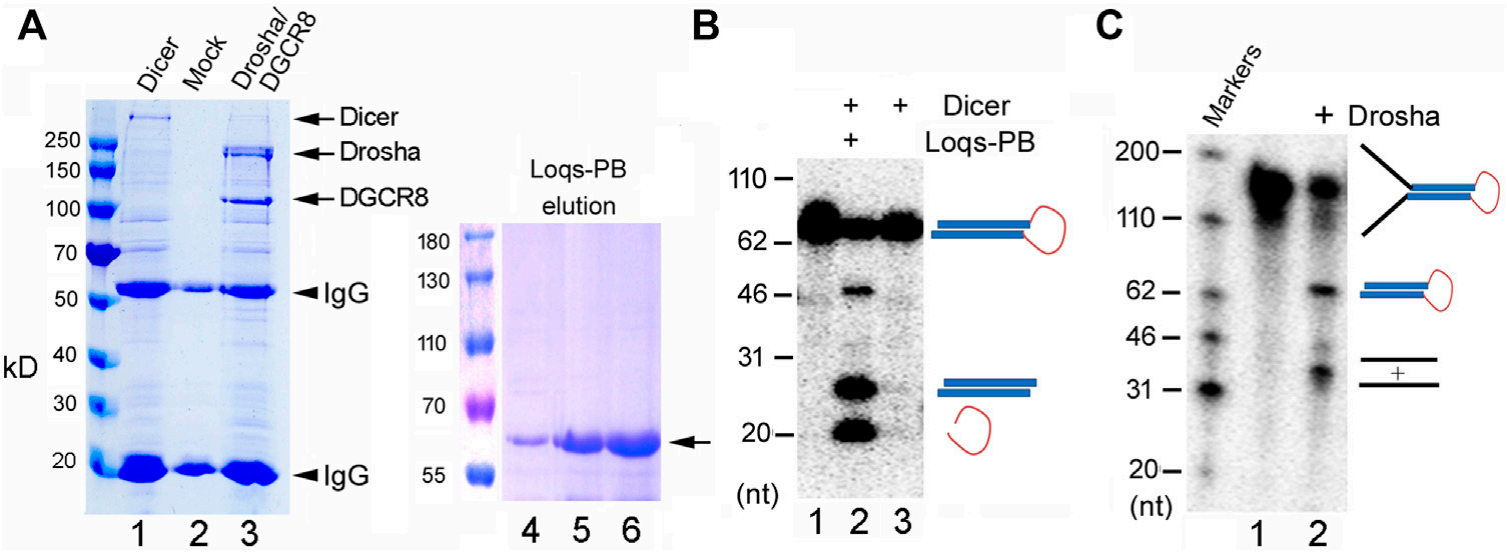
Purification and activity assays of the fruitfly miRNA processing enzymes. **(A)** Purification of recombinant Dicer, Drosha/DGCR8 from transfected 293T cells (lanes 1–3) and Loqs-PB from overexpressing bacteria (lanes 4–6). 293T cells were transfectd with plasmids expressing the FLAG-tagged Dicer or FLAG-tagged Drosha and Myc-tagged DGCR8. Following immunoprecipitation with an anti-FLAG antibody, proteins were detected by gel electrophoresis and Coomassie staining. Lane 1: The immunoprecipitate of FLAG-Dicer; lane 2: immunoprecipitate from mock-transfected cells; lane 3: immunoprecipitate of FLAG-Drosha and Myc-DGCR8; lanes 4–6: different elution fractions of His-tagged Loqs-PB after nickle beads purification. Arrows points to the expected fruitfly proteins, and arrowheads the IgG heavy and light chains. Protein markers (in kilodaltons, kD) are indicated on the left. **(B)** Dicer activity assay. The ^32^P-labeled dme-pre-miR-375 was incubated with Dicer alone or together with Loqs-PB at 37°C for 30 min, fractionated on a 12% denaturing gel, and analyzed by phosphorimaging. DNA markers with size of the individual bands in nucleotide (nt) are indicated on the left, and schematics of the substrate and cleavage products shown on the right. **(C)** Drosha processing assay. The ^32^P-labeled dme-pri-let-7 substrate was incubated with Drosha/DCGR8 (Drosha in short) at 37°C for 60 min. Samples were fractionated on a 10% denaturing gel and analyzed by phosphorimaging. Labels are the same as in **(B)**.

We had previously cloned and expressed zebrafish Dicer, Drosha, and DGCR8, and demonstrated their activities (Li et al., 2019b). For this study, therefore, we first cloned the fruitfly Dicer, Drosha, and DGCR8 into expression vectors, (co-)transfected the plasmids into 293T cells, and then purified the over-expressed proteins by immunoprecipitation. As shown in Figure 1A, we successfully expressed and isolated recombinant Dicer and Drosha/DGCR8 (Drosha hereafter for short, unless indicated otherwise). We also purified the His-tagged Loqs-PB from bacteria (Figure 1A, lanes 4–6). Consistent with previous reports (Forstemann et al., 2005; Jiang et al., 2005; Saito et al., 2005), His-Loqs-PB greatly stimulated the activity of fruitfly Dicer towards a fly pre-miRNA, dme-pre-miR-375 (Figure 1B, compare lanes 2 and 3). Fruitfly Drosha also cleaved dme-pri-let-7 to produce dme-pre-let-7 (Figure 1C, lane 2); this was the first time the fruitfly Drosha holoenzyme had been reconstituted using largely purified components. Thus, we were able to express and purify the active, zebrafish and fruitfly Dicer and Drosha RNases.

### Large-Scale Cleavage Assays by Drosha and Dicer

The biogenesis of zebrafish or fruitfly miRNAs had not been characterized in detail. Previous studies using conventional strategies to identify miRNA features required for processing examined only select human miRNAs and their mutants *in vitro* and lacked a direct reference to endogenous miRNA expression (Lee et al., 2003; Zeng and Cullen, 2003; Zeng and Cullen, 2005; Zeng et al., 2005; Han et al., 2006). To overcome the limitation, we employed a different, global approach. As an example, miRBase categorizes 355 zebrafish miRNA genes, including 285 in 56 miRNA families containing two or more family members (Kozomara et al., 2019). We arbitrarily chose a representative from each of these families, e.g., dre-let-7d-2 from the let-7 family, except two miRNAs from the dre-miR-126 family. For the remaining 70 zebrafish miRNAs we randomly selected 55 for further analysis. So altogether we examined 112 dre-pri-miRNAs for processing by zebrafish Drosha (Supplementary Table S3). Similarly, we also selected 108 dre-pre-miRNAs for processing by zebrafish Dicer, 119 dme-pri-miRNAs for fruitfly Drosha, and 120 dme-pre-miRNAs for fruitfly Dicer (Supplementary Tables S1, S2, S4, respectively). We then designed primers, synthesize DNA templates by PCR, and produced RNAs by *in vitro* transcription.

**FIGURE 2:**
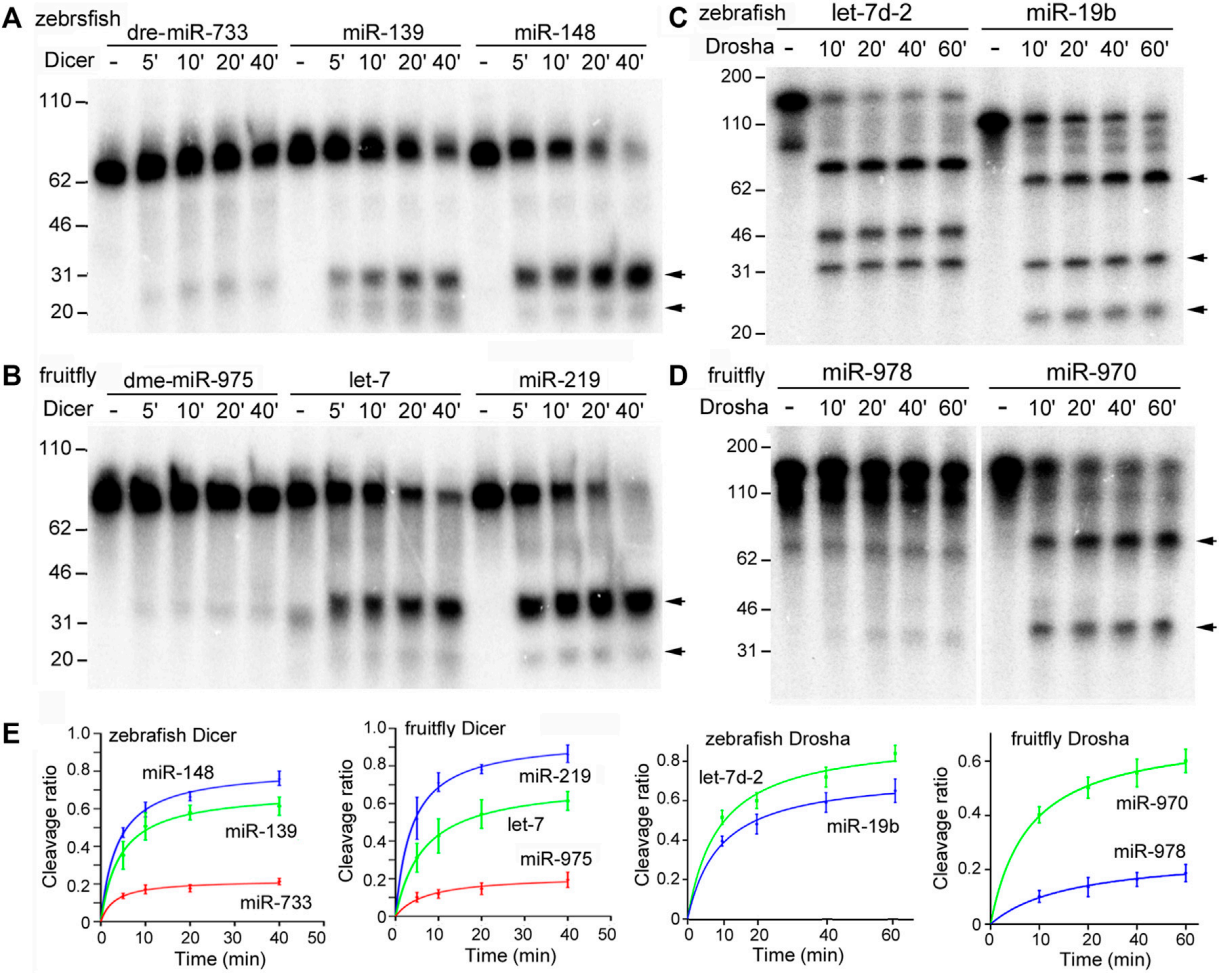
Time-course processing reactions by zebrafish and fruitfly Dicer and Drosha. **(A)** Cleavage of dre-pre-miRNAs by zebrafish Dicer. Arrows point to cleavage products. Timepoints are indicated on top of the gel image, DNA markers (in nt) shown on the left. **(B)** Cleavage of dme-pre-miRNAs by fruitfly Dicer. **(C)** Cleavage of dre-pri-miRNAs by zebrafish Drosha. **(D)** Cleavage of dme-pri-miRNAs by fruitfly Drosha. **(E)** Quantification of the data in **(A–D)**. The y-axes are the raw substrate cleavage ratios, with averages and standard deviations, plotted against the different time points in the x-axes.

**FIGURE 3:**
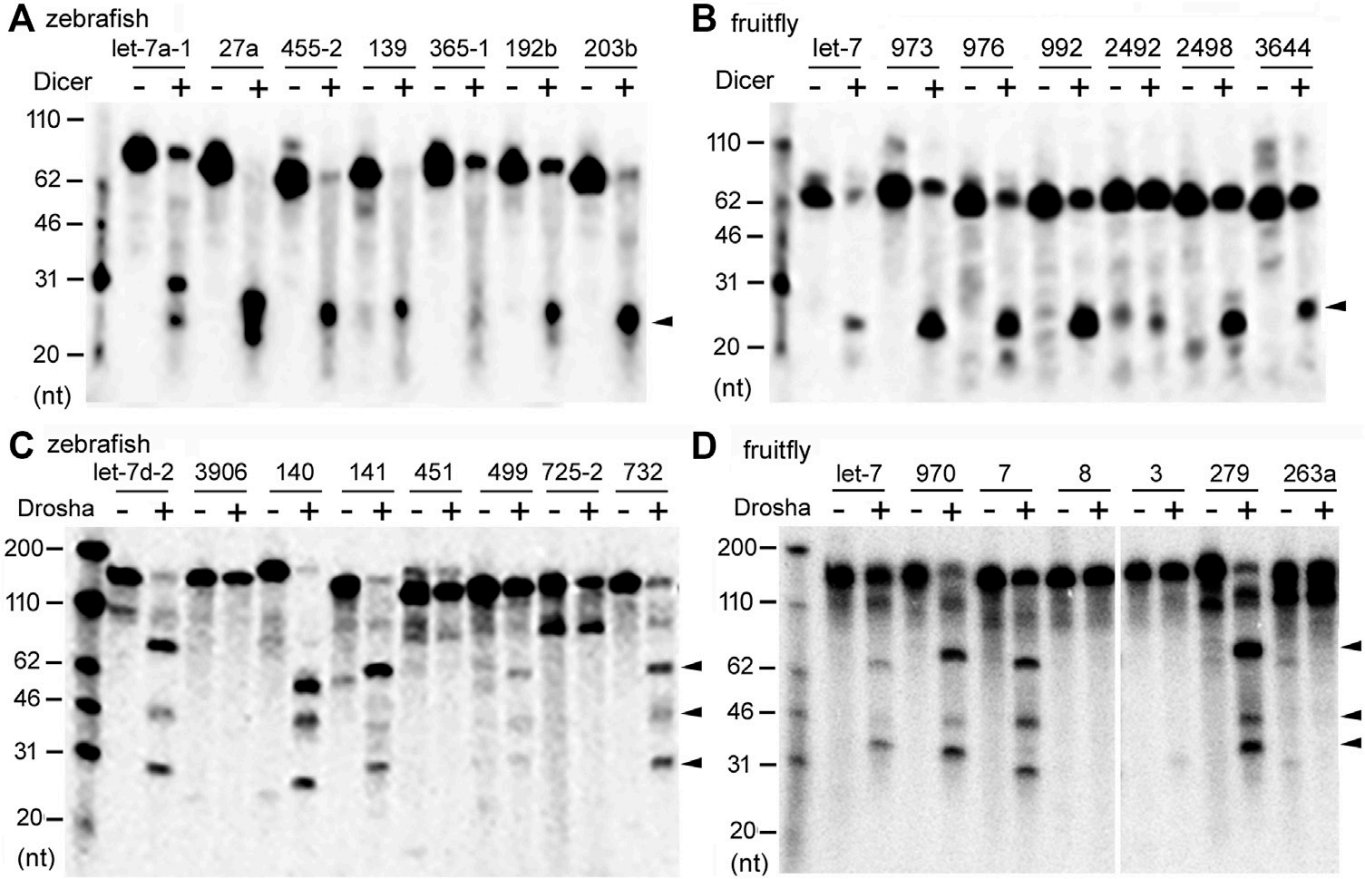
Representative processing reactions by zebrafish and fruitfly Dicer and Drosha. **(A)** Cleavage of ^32^P-labeled dre-pre-miRNAs by zebrafish Dicer. miRNA substrates are indicated on top of the gel image, DNA markers shown on the left, and the arrowhead points to the cleavage product(s). **(B)** Cleavage of dme-pre-miRNAs by fruitfly Dicer. Labeling is the same as in **(A)**. **(C)** Cleavage of dre-pri-miRNAs by zebrafish Drosha. Arrowheads point to cleavage products. **(D)** Cleavage of different dme-pri-miRNAs by fruitfly Drosha. Labeling is the same as in **(C)**.

*In vitro* Dicer and Drosha processing assays were then performed on the chosen pre-miRNAs and pri-miRNAs, respectively. Previous reports (Feng et al., 2012; Li et al., 2019a) and preliminary experiments (Figure 2) showed that results of the end-point assays matched those of the time-course assays, and that the zebrafish and fruitfly Dicer and Drosha enzymes indeed processed some substrates faster than others (Figures 2A–D, respectively). At all the timepoints tested, poor substrates were cleaved less than the good ones (Figure 2E). Subsequently, we used end-point assays and Spearman correlation analyses to compare and evaluate the cleavage efficiencies of different RNA substrates. Processing efficiency data for all the RNAs are listed in Supplementary Tables S1–S4, and results of the representative experiments shown in Figure 3. Both zebrafish and fruitfly Dicer exhibited strong cleavage activities against pre-miRNAs (Figures 3A,B). Zebrafish Drosha also cleaved most of the pri-miRNA substrates (Figure 3C; Supplementary Table S3). On the other hand, fruitfly Drosha cleaved only approximately half of the tested pri-miRNAs (Figure 3D; Supplementary Table S4). As expected, fruitfly Drosha did not digest dme-pri-miR-1006 or dme-pri-miR-1012 (Supplementary Table S4), which are derived from mirtrons known to bypass Drosha requirement (Okamura et al., 2007; Ruby et al., 2007). Most importantly, pre-miRNAs and pri-miRNAs varied in their susceptibility to Dicer and Drosha, respectively (Figures 2, 3; Supplementary Tables S1–S4). For example, Dicer cleaved dme-pre-let-7 more efficiently than dme-miR-975 (Figure 2B) and dme-pre-miR-2492 (Figure 3B), and dre-pri-let-7d-2 was the better substrate than dre-pri-miR-499, which in turn was much better than dre-pri-miR-3906 (Figure 3C). Thus, just like human Dicer and Drosha (Feng et al., 2011; Feng et al., 2012), their zebrafish and fruitfly counterparts also exhibited relative specificity by discriminating against their substrates.

### Differential Drosha Processing Correlates With Global, Endogenous miRNA Expression

What is the biological significance of the observed substrate selectivity or preferences? To answer this question, we compared our relative pre-miRNA and pri-miRNA cleavage efficiencies to genome-wide miRNA expression levels in zebrafish (GSE57169) and fruitflies (GSE163852). Relative pre-miRNA cleavage efficiencies by Dicer did not correlate with miRNA expression in zebrafish or fruitflies (Table 1), consistent with results of human Dicer (Feng et al., 2012). On the other hand, pri-miRNA cleavage by Drosha positively and significantly correlated with global miRNA expression patterns (*p* < 0.05; Table 1). Even though the correlation was weak, it nonetheless indicated that efficient Drosha processing enhances endogenous miRNA production. Thus, from fruitflies to humans, both Dicer and Drosha cleave their miRNA substrates selectively, but only the relative specificity of Drosha dominates and contributes to differential miRNA expression *in vivo*.

**TABLE 1:** Correlation between Dicer and Drosha cleavage efficiencies and global miRNA expression *in vivo*. Sample size (N), Spearman’s correlation coefficient (ρ), and *p* values are listed. Correlations with *p* < 0.05 are marked in red. Raw data are presented in Supplementary Tables S1–S4.

	miRNA expression		
	N	ρ	*p*
Zebrafish Dicer	106	0.053	0.589
Fruitfly Dicer	120	0.070	0.451
Zebrafish Drosha	110	0.247	0.009
Fruitfly Drosha	119	0.200	0.029

**FIGURE 4:**
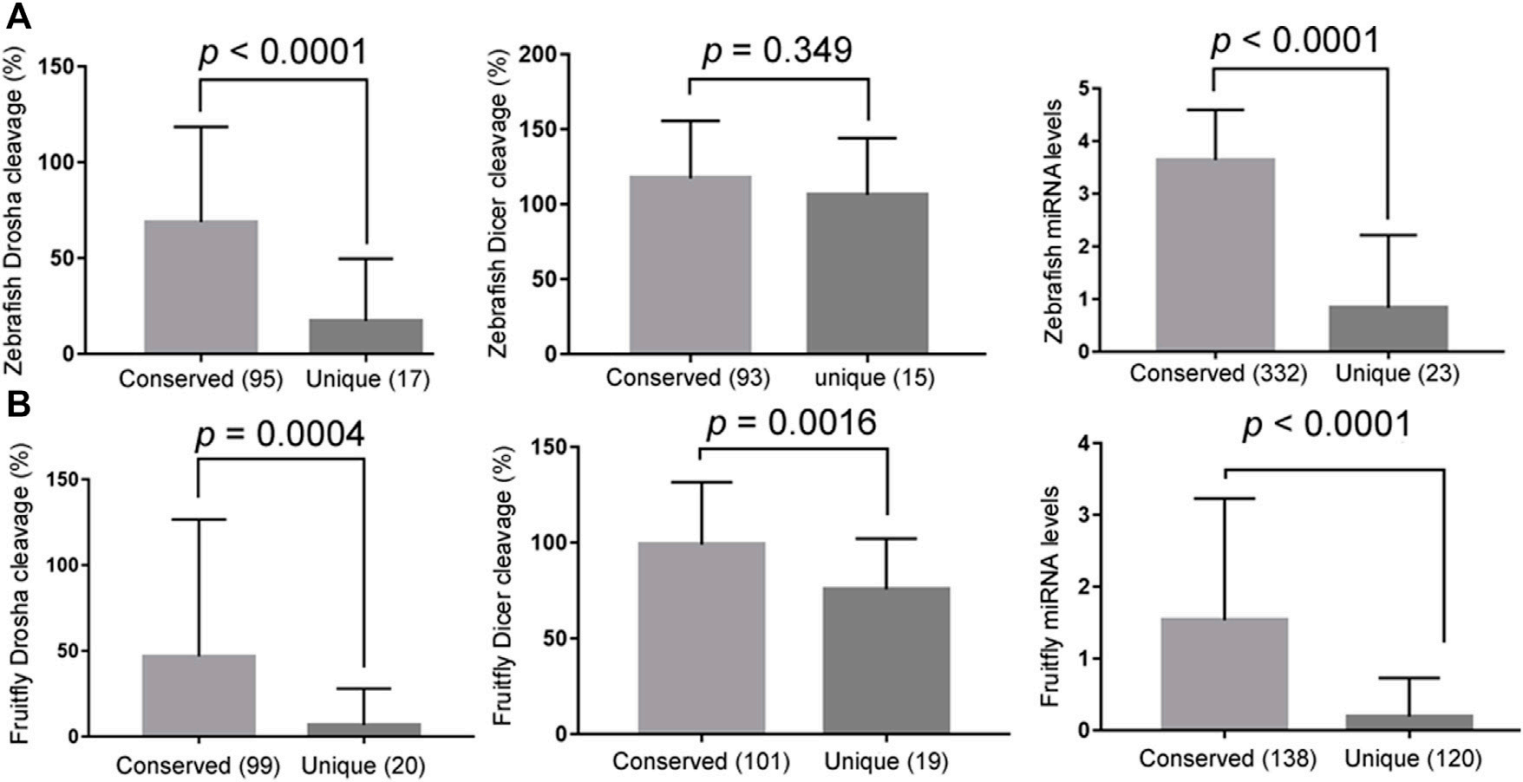
Conserved miRNAs are more efficiently processed and better expressed. **(A)** Comparing the relative Drosha cleavage efficiencies **(the left panel)**, Dicer cleavage efficiencies **(the middle panel)**, and mature miRNA expression **(the right panel)** between the conserved and unique zebrafish miRNAs (x-axes). The right panel analyzed all the miRNAs, i.e., not just the Drosha or Dicer substrates examined in this study, and the y-axis is miRNA expression values after log_10_ transformation (GSE57169). Averages and standard deviations are shown, the *P* values listed on top, and parentheses indicate the numbers of miRNAs in the categories. **(B)** Comparing the relative Drosha cleavage efficiencies **(the left panel)**, Dicer cleavage efficiencies **(the middle panel)**, and mature miRNA expression (GSE163852; **the right panel**) between the conserved and unique fruitfly miRNAs (x-axes). Labels are the same as in **(A)**.

Conserved miRNAs, i.e., those belonging to miRNA gene families, tended to have higher Drosha and, to a lesser extent, Dicer processing efficiencies than non-conserved miRNAs (Figures 4A,B, the left and middle panels, respectively; Supplementary Tables S1–S4). Likewise, conserved miRNAs were also more highly expressed in zebrafish and fruitflies (Figures 4A,B, right panels; Supplementary Tables S1–S4). These results matched those in humans (Feng et al., 2011) and suggested that conserved miRNAs have evolved to be processed efficiently, especially by Drosha, which would have facilitated their expression *in vivo*.

### Structural Features Underlying the Efficiencies of miRNA Processing

**FIGURE 5:**
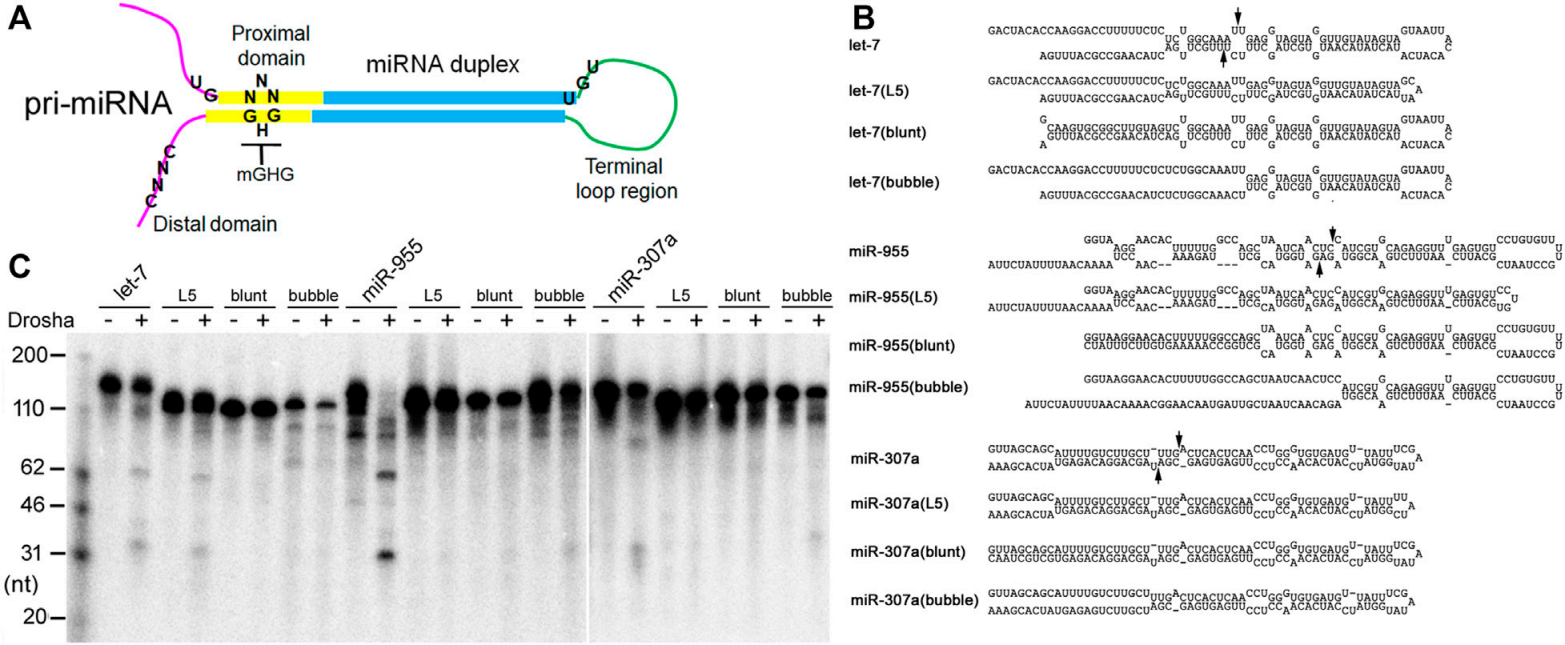
Pri-miRNA preferences by Drosha. **(A)** Schematics of an animal pri-miRNA. The important secondary structural features are shown, along with positions of the UG, UGU, mGHG, and CNNC motifs. **(B)** Predicted secondary structures of dme-pri-miRNAs and their mutants. Arrows point to Drosha cleavage sites predicted by miRBase. **(C)** Fruitfly Drosha processing of the dme-pri-miRNAs and their mutants. Positions of DNA markers are shown in the left.

How do Dicer and Drosha distinguish between substrates, and can we predict whether an RNA is a good substrate or not, or how well a miRNA is produced *in vivo*? A pri-miRNA contains a number of structural features: a pre-miRNA moiety composed of a terminal loop region and miRNA duplex, and the flanking region, further divided into the proximal domain or basal stem and distal domain (Figure 5A; Feng et al., 2011). The recently solved structures of Drosha:pri-miRNA complexes indicate that the DGCR8 subunit binds the terminal loop region and its junction with the miRNA duplex, while the Drosha subunit binds rest of the stem and the distal domain, providing a clear rationale of why these distinct RNA structures are important for processing (Jin et al., 2020; Partin et al., 2020). Variations in these structures in diverse RNAs are expected to modulate processing by Drosha and Dicer. Indeed, previous correlation analyses had shown that efficient cleavage of human pri-miRNAs requires a flexible terminal loop region and a largely helical proximal domain (Feng et al., 2011). We thus predicted the secondary structure and ΔG of the zebrafish and fruitfly pre-miRNA and pri-miRNA sub-structures (Supplementary Tables S1–S4) and then correlated the predictions to cleavage efficiencies and to mature miRNA expression in the corresponding organisms.

Table 2 shows that ΔG of the proximal domain significantly (*p* < 0.05) and negatively correlated with cleavage by both zebrafish and fruitfly Drosha, and with miRNA expression *in vivo*. This is consistent with a stem requirement: the more stable the proximal domain, hence the lower ΔG, the higher the Drosha cleavage efficiencies and the higher the miRNA maturation in zebrafish and fruitflies. ΔG of the terminal loop region at the other end positively correlated with Drosha cleavage (Table 2), indicating preferences for a single-stranded RNA conformation: the higher ΔG, the less stable the terminal loop region, the more efficient Drosha cleavage. A relaxed terminal loop region also favored fruitfly Dicer (Table 2). These results are consistent with those in humans (Feng et al., 2011; Feng et al., 2012). ΔG of the distal domain positively correlated with cleavage by fruitfly Drosha, but not by zebrafish Drosha, and it also positively correlated with global fruitfly miRNA production (Table 2). It is well-established that Drosha requires single-stranded RNA in the distal domain (Zeng and Cullen, 2005; Han et al., 2006), even though we did not identify a positive correlation between ΔG of the distal domain and human Drosha processing, either (Feng et al., 2011). An explanation is that our human and zebrafish substrates have sufficiently flexible distal domains. Lastly, a stable miRNA duplex moiety enhanced miRNA expression in zebrafish and fruitflies (*p* < 0.05, Table 2).

**TABLE 2:** Correlation between predicted RNA structures and miRNA cleavage or miRNA expression. Sample size (N), Spearman’s correlation coefficient (ρ), and *p* values are listed. Correlations with *p* < 0.05 are marked in red. Raw data are in Supplementary Tables S1–S4.

Structure (by ΔG, kcal/mol)	Dicer cleavage efficiency			Drosha cleavage efficiency			miRNA expression		
	N	ρ	*p*	N	ρ	*p*	N	ρ	*p*
dre-pre-miRNA	108	−0.022	0.825				106	−0.413	<0.0001
miRNA duplex	108	−0.092	0.344				106	−0.382	0.0001
Terminal loop region	108	0.168	0.081				106	−0.066	0.499
dre-pri-miRNA				112	−0.145	0.128	110	−0.360	0.0001
Distal domain				112	0.082	0.485	110	−0.090	0.352
Proximal domain				112	−0.301	0.001	110	−0.345	0.0002
miRNA duplex				112	−0.165	0.083	110	−0.357	0.0001
pre-miRNA				112	−0.049	0.606	110	−0.324	0.0006
Terminal loop region				112	0.202	0.033	110	0.118	0.219
dme-pre-miRNA	120	−0.058	0.531				120	−0.180	0.049
miRNA duplex	120	−0.161	0.080				120	−0.202	0.027
Terminal loop region	120	0.207	0.023				120	0.080	0.384
dme-pri-miRNA				119	−0.039	0.670	119	−0.119	0.198
Distal domain				119	0.299	0.001	119	0.275	0.003
Proximal domain				119	−0.202	0.028	119	−0.234	0.010
miRNA duplex				119	−0.189	0.040	119	−0.193	0.035
pre-miRNA				119	−0.078	0.401	119	−0.168	0.067
Terminal loop region				119	0.213	0.020	119	0.080	0.390

Substrate requirements of fruitfly Drosha had not been examined previously. Thus, to further confirm the RNA secondary structure preferences of fruitfly Drosha, we introduced mutations to dme-pri-let-7, dme-pri-miR-955, and dme-pri-miR-307a. As shown in Figure 5B, the “L5” mutants contained a smaller terminal loop region, the “blunt” mutants contained a basepaired distal domain, while the “bubble” mutants contained a prominently single-stranded proximal domain. Drosha cleaved all these mutants less efficiently than the wildtype pri-miRNAs (Figure 5C). The same results have been obtained with zebrafish Drosha (Li et al., 2019b). Together, our biochemical and bioinformatics analyses demonstrated that miRNA processing mechanisms are conserved from fruitflies to humans, that a stem feature in the proximal domain and flexibility in the terminal loop region are crucial determinants of Drosha processing, and that the proximal domain modulates genome-wide miRNA maturation *in vivo*.

### Impacts of Sequence Motifs on miRNA Processing and Expression

**FIGURE 6:**
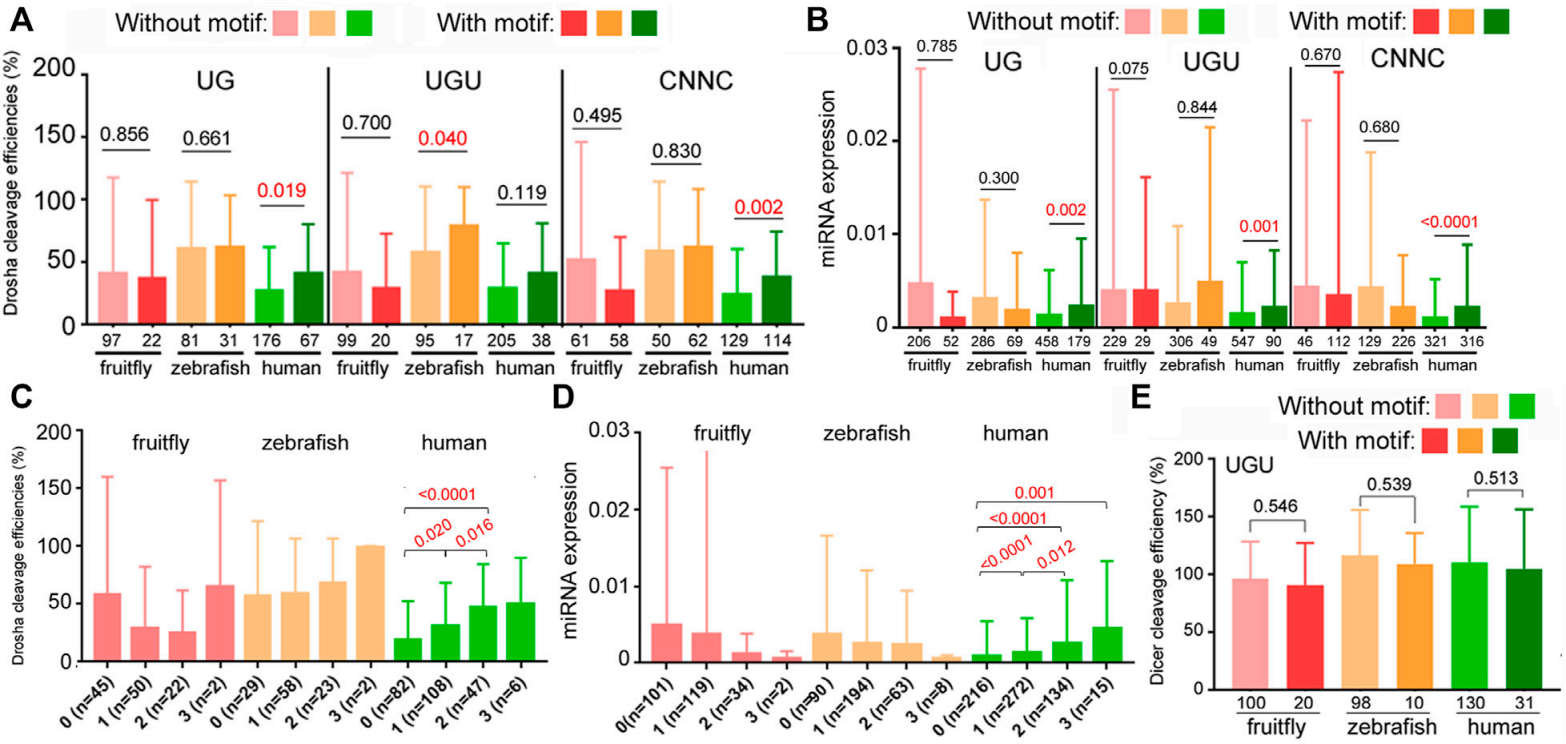
Contributions by UG, UGU, and CNNC motifs to Drosha processing and miRNA expression. **(A)** Comparison of relative Drosha cleavage efficiencies (y-axis) of fruitfly, zebrafish, and human pri-miRNAs with or without the UG, UGU, or CNNC motif (listed on top). Different colors represent different species (x-axis). Columns on the left represent miRNAs without the sequence motif, those on the right with the motif. Integers underneath the columns indicate numbers of miRNAs in the categories. Averages and standard deviations are shown, and the *P* values shown above the columns, with those <0.05 in red. **(B)** Normalized expression (y-axis) of fruitfly, zebrafish, and human miRNAs (x-axis) with or without the UG, UGU, or CNNC motif (on top). Labels are the same as in **(A)**. **(C)** Comparison of relative Drosha cleavage efficiencies (y-axis) of fruitfly, zebrafish, and human pri-miRNAs with 0, 1, 2, or 3 of the UG, UGU, and/or CNNC motifs (x-axis). Labeling is the same as in **(A)**. The various categories are indicated at the bottom, with numbers of the miRNAs listed in parentheses. **(D)** Normalized expression (y-axis) of fruitfly, zebrafish, and human miRNAs with 0, 1, 2, or 3 of the UG, UGU, and/or CNNC motifs (x-axis). Labels are the same as in **(C)**. **(E)** Comparison of relative Dicer cleavage efficiencies (y-axis) of fruitfly, zebrafish, and human pre-miRNAs with or without the UGU motif. Different colors represent different species (x-axis). Columns on the left represent miRNAs without the sequence motif, those on the right with the motif. Integers underneath the columns indicate numbers of miRNAs in the categories. Averages and standard deviations are shown, and the *P* values indicated above the columns.

Besides the aforementioned structural features, subsets of pri-miRNAs also contain one or more sequence preferences, such as UG, UGU, mGHG, and CNNC, at different positions, which might help processing and specify Drosha cleavage sites (Figure 5A; Auyeung et al., 2013; Fang and Bartel, 2015; Kwon et al., 2019). These sequences had been mostly characterized using variants of select human miRNAs, so it is unknown how they contribute to endogenous miRNA processing and expression in humans or other species. To evaluate the roles of these sequences in authentic miRNAs at the genome level, we first examined how the presence of UG, UGU, or CNNC affected relative miRNA processing efficiencies and miRNA expression in fruitflies, zebrafish, and humans. The UG motif was deemed missing in insect miRNAs (Auyeung et al., 2013; Kwon et al., 2019), although we found a similarly small number of fruitfly miRNAs containing the UG or UGU motif (Figures 6A,B). The most consistent pattern was observed with human miRNAs that contained the UG or CNNC sequence being cleaved by Drosha more efficiently as well as expressed at a higher level than those without (Figures 6A,B, *p* < 0.05; Supplementary Table S5). For fruitfly and zebrafish miRNAs, the UG and CNNC motifs had no significant impact on Drosha processing or miRNA expression levels *in vivo* (Figures 6A,B; Supplementary Tables S3, S4). The UGU motif had variable influences on zebrafish and human Drosha processing and miRNA expression (Figures 6A,B). We also tested if the presence of one or more of these three sequences might improve processing and miRNA expression. We observed an increase for only human miRNAs (Figures 6C,D; Supplementary Tables S3–S5). The UGU motif had no effect on pre-miRNA processing by Dicer (Figure 6E; Supplementary Tables S1, S2, S5).

We then examined the contribution by mGHG. Kwon et al. (2019) assigned mGHG scores to different human miRNAs, and based on the same formula we calculated the scores for fruitfly and zebrafish miRNAs. In all three organisms the relative Drosha cleavage efficiencies as well as miRNA expression correlated positively and significantly with mGHG scores (Table 3). Thus, mGHG is an evolutionarily conserved feature in pri-miRNAs that enhances and regulates Drosha processing and miRNA expression *in vivo*. Our analyses did not examine how mGHG or other motifs affected cleavage site selection.

**TABLE 3:** Correlation between mGHG scores and miRNA cleavage or miRNA expression. Sample size (N), Spearman’s correlation coefficient (ρ), and *P* values are listed. Raw data are presented in Supplementary Tables S3-S5.

Species	Drosha cleavage efficiency			miRNA expression		
	N	ρ	*p*	N	ρ	*p*
Fruitfly	91	0.283	0.006	157	0.215	0.007
Zebrafish	91	0.261	0.012	283	0.128	0.032
Human	169	0.258	0.001	459	0.189	<0.0001

## Discussion

In this study we examined the cleavage of hundreds of zebrafish and fruitfly pre-miRNAs and pri-miRNAs by Dicer and Drosha, respectively. We showed that both Dicer and Drosha discriminate their substrates *in vitro*, but only the preference of Drosha correlates with global, differential miRNA expression *in vivo*. We identified distinct structural features and sequence motifs that enhance miRNA processing and miRNA biogenesis at the genome-wide scale in animal species. We concluded that miRNA processing mechanisms are conserved from fruitflies to humans, so is the functional consequence of differential cleavage by Drosha, hence relative specificity in general, in regulating miRNA expression.

When an E has many interacting partners or substrates, it is reasonable to assume that it will not treat them equally, although explicit reports are scant. As Figures 2, 3 and Supplementary Tables S1–S4 show, zebrafish and fruitfly Dicer and Drosha cleave their respective miRNA substrates with varying efficiencies. Yet in both organisms only differential cleavage, i.e., relative specificity, by Drosha significantly and positively correlates with endogenous miRNA expression (Table 1). While the correlation coefficients are low, we note that Drosha cleavage efficiencies were measured *in vitro*, using substrates much shorter than endogenous pri-miRNAs, contributions by many proteins known to influence the processing of individual miRNAs were ignored, and noises in the processing and miRNA expression data always lead to underestimation of the coefficients (Csardi et al., 2015). Finding a significant correlation explainable by the biochemical mechanism of miRNA processing was, therefore, never a given. Most importantly, our present, comparative results are consistent with those obtained for human Dicer and Drosha (Feng et al., 2011; Feng et al., 2012). As shown in humans (Feng et al., 2011), conserved zebrafish and fruitfly miRNAs are also better Drosha substrates and more highly expressed, indicating miRNA processing and, hence, production have been under selection (Figures 4A,B). Thus, by examining a large number of substrates at once, we aimed for the general trend and scope, whereas consistency of the results across species indicates minimal sampling or data bias. Together, these data show that both relative specificity in miRNA processing and the relative contribution by Drosha and Dicer to differential miRNA expression are conserved.

To elucidate how relative specificity arises, we showed that the proximal domain and terminal loop region significantly impact how efficiently the zebrafish and fruitfly Drosha cleaves pri-miRNAs, and that a relatively large terminal loop enhances fruitfly Dicer activity (Table 2). As the human counterparts have the same preferences (Feng et al., 2011; Feng et al., 2012), how Drosha and Dicer interact with and select their substrates has been conserved through evolution, even though the fruitfly and human orthologs share only 37–50% identity (Li et al., 2019b). A requirement for single-stranded RNA conformation has also been identified for the distal domain in human pri-miRNAs (Zeng and Cullen, 2005; Han et al., 2006), although correlation analyses reveal only a significant link between the distal domain and relative cleavage efficiencies or miRNA expression in fruitflies (Table 2), but not in humans (Feng et al., 2011) or zebrafish (Table 2). This may be partly because we designed those substrates with an open distal domain already. Compared to the results in humans (Feng et al., 2011), we also found miRNA expression in zebrafish and fruitflies significantly associates with a stable proximal domain, although not with the terminal loop (Table 2). Because miRNAs and miRNAs* have isoforms, and their 5′ and 3′ ends are not as well defined in zebrafish and fruitflies as in humans, our lesser ability to demarcate the terminal loop regions might be a compounding factor.

In addition to the secondary structure features, we also examined the roles of sequence motifs first identified in certain human pri-miRNAs, as it had not been determined previously whether these motifs influence endogenous miRNA biogenesis or whether they function in species other than humans (Auyeung et al., 2013; Fang and Bartel, 2015; Kwon et al., 2019). Figure 6 shows the positive effects of the UG and CNNC motifs on human Drosha processing and miRNA expression. The mGHG motif is more prevalent in animal pri-miRNAs and facilitates Drosha processing as well as miRNA expression from fruitflies to humans (Table 3). The basic architecture of animal pri-miRNAs is similar, but species-specific miRNA biogenesis has been suggested for worms and, to a lesser extent, fruitflies (Auyeung et al., 2013), raising the question of how well miRNA processing is conserved. Our studies show for the first time that fruitflies prefer the same secondary structures and mGHG motif for pri-miRNA processing and miRNA expression (Tables 2, 3; Figure 5), indicating the general conservation of miRNA processing mechanisms.

It has been proposed that Drosha, an essential RNase in the production of canonical miRNAs, by differentially cleaving its myriad substrates, further regulates genome-wide miRNA expression (Feng et al., 2011; Conrad et al., 2014, and this study). Interestingly, such a regulatory role is not ascribed to Dicer, which also cleaves some pre-miRNAs better than others. Once transcribed, pri-miRNAs may have multiple fates, serving as intermediates for miRNAs, mRNAs, or other non-coding RNAs (Bartel, 2018). As Drosha can digest pri-miRNAs co-transcriptionally (Morlando et al., 2008), and the cleavage of pri-miRNAs initiates the irreversible step in miRNA biogenesis, it makes sense that the differential cleavage activity of Drosha would dominate. And compared to Dicer, Drosha recognizes a much larger RNA substrate, with its activity more sensitive to the combined RNA features. Consistent with this explanation, pri-miRNAs differ greatly in their susceptibility to Drosha *in vitro*, whereas Dicer was able to cleave all the pre-miRNAs, with smaller variations in efficiencies (Figure 3; Supplementary Tables S1–S4). Nonetheless, Dicer may still play a secondary, global role, or by acting with specific RNA-binding proteins modulate the expression of individual miRNAs. We conclude that differential processing serves an important function in regulating miRNA expression, and the principle of relative specificity is evolutionarily conserved.

## Data Availability

The datasets presented in this study can be found in online repositories. The names of the repository/repositories and accession number(s) can be found below: https://www.ncbi.nlm.nih.gov/geo/, GSE163852.

## Abbreviations

miRNA, microRNA; RNase, ribonuclease; pri-miRNA, primary microRNA; pre-miRNA, precursor microRNA; nt, nucleotide.
